# Longitudinal study on the relationship between extracellular water distribution changes and muscle mass in severe sarcopenia patients using multi-frequency bioelectrical impedance analysis combined with phase angle measurements

**DOI:** 10.1007/s41999-025-01383-w

**Published:** 2026-01-03

**Authors:** Rong Chen, Zhaohui Xu, Hongwei Shi, Tengfei Ma, Pengfei Li, Rong Yuan, Chunfeng Liu

**Affiliations:** Department of Critical Care Medicine, Rudong People’s Hospital, Nantong, 226400 China

**Keywords:** Sarcopenia, Bioelectrical impedance analysis, Phase angle, Muscle mass, Body composition, Aging

## Abstract

**Aim:**

This study aimed to explore whether changes in extracellular water distribution and phase angle can serve as early markers of muscle loss in patients with severe sarcopenia.

**Methods:**

A 12-month prospective study enrolled 128 severe sarcopenia patients (72 M, 56F; mean age 74.3 ± 6.8y), with regular multi-frequency bioelectrical impedance analysis (5–500 kHz) and phase angle measurement at 50 kHz. Body composition, physical function, and inflammation markers were periodically assessed.

**Results:**

Of the 128 enrolled participants, 112 (87.5%) completed the study. Muscle mass decreased significantly over 12 months, with the most rapid loss occurring between months 3–6. Phase angle also declined consistently, and a drop of ≥ 0.3° over three months effectively predicted accelerated muscle loss. Changes in bioimpedance parameters appeared nearly three weeks before detectable muscle loss. The extracellular water-to-total body water ratio rose progressively and correlated strongly with muscle mass reduction. Limb regions showed more notable deterioration than the trunk.

**Conclusion:**

Multi-frequency bioelectrical impedance analysis combined with phase angle measurement offers a non-invasive and sensitive approach for early detection of muscle mass loss in severe sarcopenia. Shifts in electrical parameters and water distribution precede measurable muscle decline, highlighting a potential window for timely intervention.

## Key summary points

**Aim:**

We aimed to find early signs of severe sarcopenia by tracking changes in body water distribution and muscle mass over time.

**Findings:**

- A measure called "phase angle" dropped steadily over one year and strongly predicted future muscle loss.
- The ratio of extracellular water in the body also rose significantly as patients lost muscle.
- These electrical changes appeared weeks before muscle loss became obvious, especially in the arms and legs.

**Message:**

A simple, non-invasive test that combines phase angle and water distribution measurements can help detect sarcopenia earlier-giving doctors a critical window to act before severe muscle wasting occurs.

## Introduction

Sarcopenia, a progressive skeletal muscle disorder characterized by accelerated loss of muscle mass, strength, and function, has emerged as a critical health concern affecting both aging populations and critically ill patients [1]. Recent epidemiological studies indicate that the global prevalence of sarcopenia ranges from 10 to 40% in adults aged 60 years and older, with severe cases comprising approximately 10–20% of affected individuals [2]. The condition significantly impacts patient mortality, with recent meta-analyses revealing a 2.5-fold increased risk of mortality in severe sarcopenia patients compared to those without the condition [3].

The pathophysiology of severe sarcopenia involves complex interactions between muscular, metabolic, and fluid balance systems. Recent research has highlighted the crucial role of extracellular water distribution in muscle function and mass maintenance [4]. Alterations in fluid homeostasis, particularly in the extracellular compartment, have been increasingly recognized as both a marker and potential contributor to muscle mass deterioration [5]. Evidence suggests that changes in extracellular water distribution precede clinically detectable muscle loss, potentially offering a window for early intervention [6].

Traditional assessment methods for monitoring sarcopenia progression, such as dual-energy X-ray absorptiometry (DXA) and magnetic resonance imaging (MRI), while accurate, present limitations in terms of cost, accessibility, and feasibility for frequent monitoring [7]. These constraints have prompted the exploration of alternative assessment techniques that could provide reliable, real-time information about disease progression and treatment response [8].

Multi-frequency bioelectrical impedance analysis (MF-BIA) has recently gained attention as a promising non-invasive technique for body composition assessment [9]. This method offers distinct advantages over conventional approaches, including its ability to differentiate between intracellular and extracellular water compartments [10]. When combined with phase angle measurements, MF-BIA provides valuable insights into cellular health and tissue quality [11]. Recent studies have demonstrated strong correlations between phase angle measurements and muscle function in various clinical populations [12].

The relationship between fluid distribution patterns and muscle mass changes in severe sarcopenia remains incompletely understood, particularly in the context of disease progression over time [13]. While cross-sectional studies have established associations between body fluid compartments and muscle mass [14], longitudinal data examining the dynamic relationship between these parameters is limited [15]. Understanding these temporal relationships could potentially reveal early markers of disease progression and guide therapeutic interventions [16].

The clinical significance of extracellular water distribution in severe sarcopenia has been further emphasized by recent findings linking fluid shifts to inflammatory processes and metabolic dysfunction [17]. These connections suggest potential therapeutic targets and highlight the importance of comprehensive monitoring approaches [18]. Additionally, emerging evidence indicates that alterations in extracellular fluid distribution may serve as early indicators of muscle quality deterioration, preceding changes in muscle mass and function [19].

The purpose of this longitudinal study is to investigate the dynamic relationship between extracellular water distribution changes and muscle mass in severe sarcopenia patients using MF-BIA combined with phase angle measurements, aiming to enhance understanding of disease progression patterns and improve monitoring strategies.

## Methods

### Study design and participants

This prospective longitudinal study was conducted between January 2023 and January 2024 at the Department of Critical Care Medicine, Rudong People’s Hospital, Nantong City, China, a tertiary care teaching hospital. The study protocol was reviewed and approved by the Institutional Review Board of Rudong People’s Hospital (Approval No. [2021] Lun Shen No. 71). Written informed consent was obtained from all participants or their legal representatives in accordance with the Declaration of Helsinki.

All participants were consecutively recruited from the inpatient wards of the Department of Geriatrics and Critical Care Medicine at Rudong People’s Hospital. All patients were hospitalized at the time of enrollment, and baseline assessments were carried out within the first 48–72 h after admission to avoid confounding from prolonged hospitalization. Prior to admission, all participants were living at home independently, and none were residents of nursing homes or long-term care institutions.

Patients were admitted for a range of acute or subacute medical problems, including exacerbations of chronic cardiopulmonary diseases (e.g., heart failure, COPD), community-acquired infections, functional decline with reduced oral intake, and subacute rehabilitation needs. All patients were clinically stable at the time of baseline assessment, and none were admitted for trauma or major surgery.

Eligible participants were aged 65–85 years and fulfilled the European Working Group on Sarcopenia in Older People 2 (EWGSOP2) diagnostic criteria for severe sarcopenia. Severe sarcopenia required the coexistence of: (1) low muscle strength (grip strength < 27 kg for men and < 16 kg for women), (2) low muscle quantity, and (3) low physical performance (gait speed < 0.8 m/s) [19]. Low muscle quantity was defined using the EWGSOP2 BIA-based cut-offs, with appendicular skeletal muscle mass index (ASM/height^2^) < 7.0 kg/m^2^ for men and < 5.5 kg/m^2^ for women [20]. Appendicular skeletal muscle mass (ASM) was estimated using the validated equation proposed by Sergi et al. [21]:

$${\text{ASM }}\left( {{\mathrm{kg}}} \right) = \left( {0.401 \times {\mathrm{height}}^{2} /{\text{resistance at }}50\,{\mathrm{kHz}}} \right) + \left( {3.825 \times {\mathrm{sex}}} \right) - \left( {0.071 \times {\mathrm{age}}} \right) + 5.102,$$

where sex = 1 for men and 0 for women. This equation was applied consistently for all analyses.

Exclusion criteria included acute cardiovascular events, severe renal insufficiency (eGFR < 30 mL/min/1.73 m^2^), active malignancy, electronic implants, significant edema or ascites, participation in other clinical trials, inability to complete follow-up, or cognitive impairment precluding informed consent.

Of the 156 patients screened, 128 met all eligibility criteria and were enrolled (82% inclusion rate). The high inclusion rate reflected the recruitment of hospitalized patients who had already undergone routine inpatient nutritional and functional screening, allowing efficient identification of individuals with severe sarcopenia.

### Clinical assessment and data collection

Baseline assessments were conducted by trained personnel using standardized procedures. Demographic data, medical history, and current medications were obtained through structured interviews and review of electronic medical records. Height was measured to the nearest 0.1 cm using a wall-mounted stadiometer, and body weight was recorded to the nearest 0.1 kg using a calibrated digital scale. Body mass index (BMI) was calculated as weight (kg) divided by height squared (m^2^).

Physical performance was evaluated using gait speed and grip strength. Gait speed was measured over a 4-m walkway at usual pace, with the faster of two trials recorded. Grip strength was measured using a hydraulic dynamometer (Jamar Plus+, Patterson Medical, USA). Three trials were performed for each hand with the participant seated and the elbow flexed at 90°, and the highest value was used for analysis.

### Bioelectrical impedance analysis

Body composition was assessed using a multi-frequency bioelectrical impedance analyzer (Medical Systems Inc., USA). The device was calibrated daily. Measurements were taken with participants in a supine position on a non-conductive surface, with arms abducted 30° and legs separated 45°. After cleaning the skin, four adhesive electrodes were placed on the right wrist and ankle.

Impedance values were recorded at 5, 50, 100, 200, and 500 kHz. Phase angle (PhA) was measured at 50 kHz, ASM was calculated using the Sergi et al. [21] equation, and SMI (ASM/height^2^) was derived accordingly. For all primary analyses, low muscle mass was defined exclusively using EWGSOP2 SMI cut-offs (< 7.0 kg/m^2^ for men; < 5.7 kg/m^2^ for women) were applied only in secondary sensitivity analyses, and did not influence participant selection or primary outcomes [22].

To minimize hydration-related measurement variability, all assessments were performed between 8:00–10:00 AM after an overnight fast, with avoidance of caffeine, alcohol, and vigorous physical activity for 24 h prior to testing.

### Laboratory measurements

Fasting blood samples were collected in the morning and processed within two hours. Biochemical analyses included serum creatinine, albumin, total protein, electrolytes, C-reactive protein (CRP), and interleukin-6 (IL-6). Hormonal assessments included insulin-like growth factor-1 (IGF-1) and 25-hydroxyvitamin D. All laboratory procedures adhered to external quality assurance standards.

### Nutritional assessment

Nutritional status was evaluated using the Mini Nutritional Assessment-Short Form (MNA-SF). Dietary intake was documented through a three-day food diary and analyzed using Nutritionist Pro™ software (Axxya Systems, USA). Total caloric intake, protein consumption, and micronutrient levels were compared with age-specific recommended dietary allowances.

### Follow-up protocol

Participants were followed for 12 months, with visits scheduled at baseline, monthly during the first three months, and quarterly thereafter. At each follow-up visit, all assessments were repeated under identical standardized conditions. Interim changes in medication, dietary intake, and physical activity were recorded.

### Statistical analysis

Statistical analyses were performed using SPSS version 28.0 (IBM Corp., Armonk, NY, USA). Data normality was assessed through visual inspection of histograms and the Shapiro–Wilk test. Continuous variables were expressed as mean ± standard deviation or median (interquartile range) based on their distribution. Categorical variables were presented as frequencies and percentages.

Temporal changes in muscle mass and fluid distribution were analyzed using linear mixed-effects models with random intercepts and slopes. The models included time as a continuous variable and relevant covariates including age, gender, and baseline clinical characteristics. The relationship between phase angle measurements and muscle mass changes was evaluated using Pearson or Spearman correlation coefficients, as appropriate.

Multiple regression analysis was employed to identify predictors of muscle mass loss, with variables selected based on clinical relevance and univariate analysis results. The predictive value of phase angle measurements was assessed through receiver operating characteristic (ROC) curve analysis. Statistical significance was set at *P* < 0.05, with Bonferroni correction applied for multiple comparisons.

Sample size calculation was based on detecting a clinically meaningful difference in muscle mass change (defined as 0.5 kg) over the study period, with 80% power and a two-sided α of 0.05. Accounting for an anticipated dropout rate of 20%, the target enrollment was 128 participants.

## Results

### Study population characteristics

A total of 156 hospitalized patients with clinically suspected severe sarcopenia were initially screened at the Department of Geriatrics and Critical Care Medicine, Rudong People’s Hospital. Twenty-eight patients were excluded, 12 did not meet the EWGSOP2 diagnostic criteria, 9 declined to participate, and 7 met exclusion criteria such as severe renal impairment, active malignancy, or implanted electronic devices. Consequently, 128 patients were enrolled and completed baseline assessments. During the 12-month follow-up, 112 participants (87.5%) completed all scheduled evaluations. Sixteen participants discontinued the study: seven due to acute illness requiring prolonged hospitalization, five withdrew consent, and four died.

**Fig. 1:**
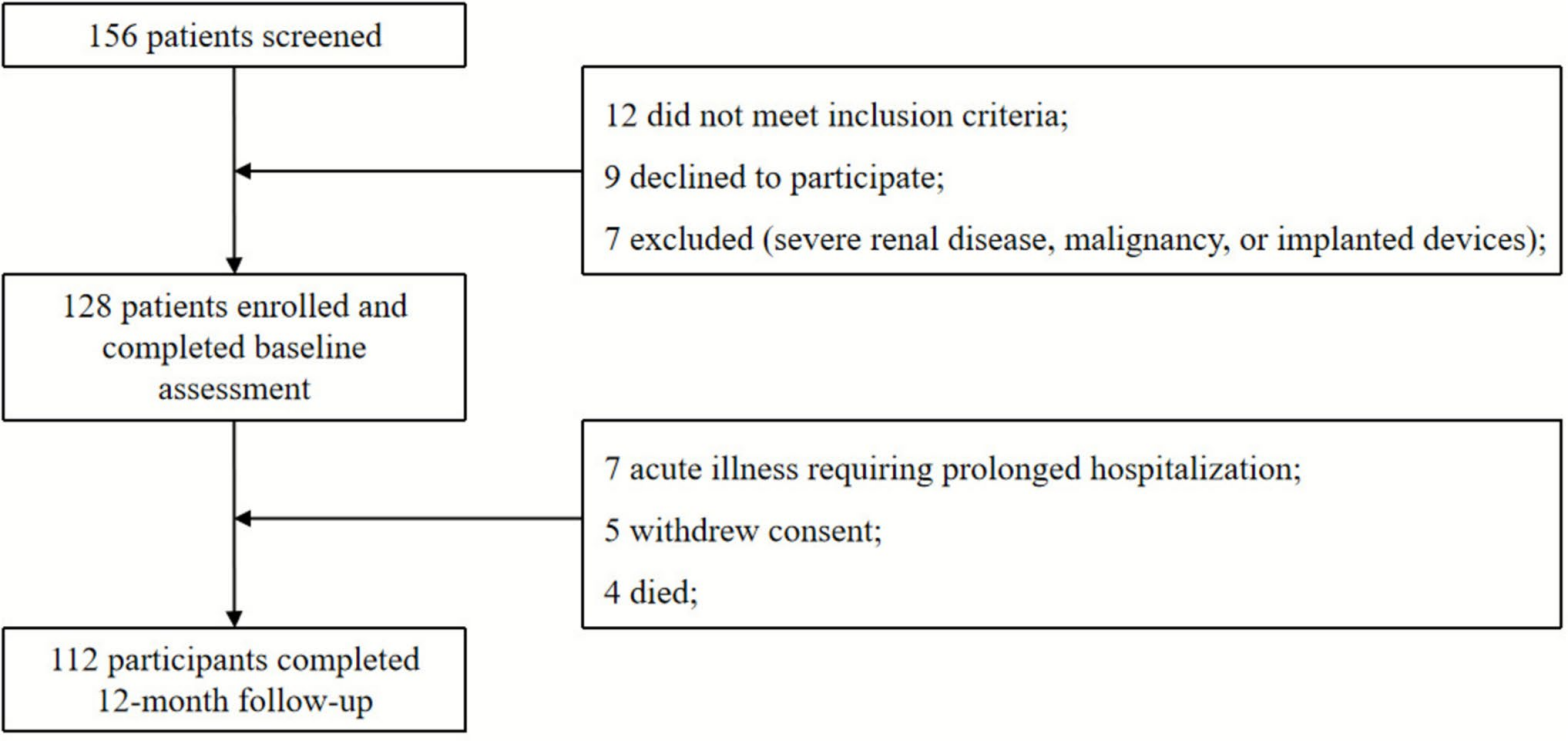
Study flowchart

The study population comprised 72 males (56.3%) and 56 females (43.7%), with a mean age of 74.3 ± 6.8 years. Baseline characteristics of the study population are presented in Table 1. No significant differences were observed in baseline characteristics between participants who completed the study and those who withdrew (*P* > 0.05 for all comparisons) (Fig. 1).

**Table 1:** Baseline characteristics of study participants (N = 128)

Characteristic	Total Population (N = 128)	Males (n = 72)	Females (n = 56)	P-value
Age (years)	74.3 ± 6.8	73.8 ± 7.1	74.9 ± 6.4	0.326
BMI (kg/m^2^)	23.1 ± 3.2	23.4 ± 3.1	22.7 ± 3.3	0.218
Skeletal muscle index (kg/m^2^)	6.2 ± 0.8	6.8 ± 0.7	5.5 ± 0.6	< 0.001
Handgrip strength (kg)	18.3 ± 4.6	21.4 ± 3.8	14.3 ± 2.7	< 0.001
Gait speed (m/s)	0.70 ± 0.15	0.72 ± 0.16	0.68 ± 0.14	0.142
Phase angle (degrees)	4.8 ± 0.9	5.1 ± 0.8	4.4 ± 0.9	< 0.001
Albumin (g/dL)	3.8 ± 0.4	3.9 ± 0.4	3.7 ± 0.4	0.156
CRP (mg/L)	3.2 (1.8–5.7)	3.0 (1.7–5.5)	3.4 (1.9–5.9)	0.284

Values are presented as mean ± SD or median (interquartile range)

### Temporal changes in body composition

**Fig. 2:**
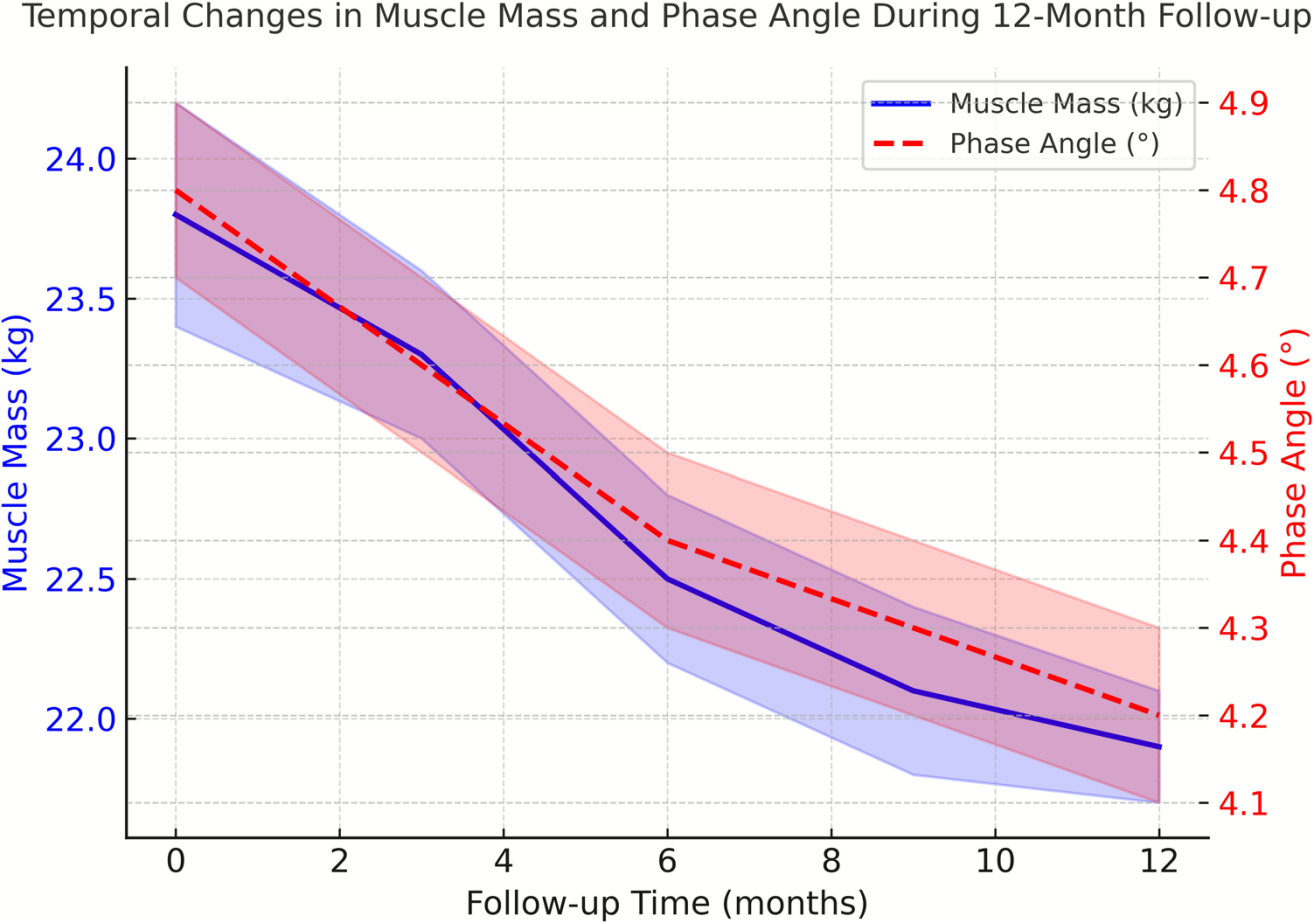
Temporal changes in muscle mass and phase angle during 12-month follow-up. Changes in muscle mass (solid blue line, expressed in kilograms [kg]) and phase angle (red dashed line, expressed in degrees [°]) were recorded in patients with severe sarcopenia (N = 112). Values represent mean ± standard error of the mean (SEM); shaded areas indicate the 95% confidence interval. A decline in phase angle appeared earlier than the subsequent measurable loss of muscle mass, suggesting that alterations in cellular integrity and electrical properties precede detectable changes in muscle quantity (P < 0.001)

Significant changes in body composition were observed over the 12-month follow-up period. Mean muscle mass showed a progressive decline from baseline (Fig. 2), with an average reduction of 1.84 ± 0.62 kg (95% CI 1.72–1.96, *P* < 0.001) over the study period. The rate of muscle loss was non-linear, with accelerated deterioration observed between months 3 and 6 (mean loss 0.76 ± 0.28 kg, *P* < 0.001), followed by a more gradual decline in the subsequent months.

Skeletal muscle index decreased by 0.71 ± 0.31 kg/m^2^ (95% CI 0.65–0.77, *P* < 0.001) over the 12-month period. The decline was more pronounced in male participants (0.82 ± 0.33 kg/m^2^) compared to females (0.58 ± 0.27 kg/m^2^, *P* = 0.003). Multivariate analysis revealed that baseline phase angle (β = − 0.45, *P* < 0.001) and inflammatory markers (β = 0.29, *P* = 0.002) were significant independent predictors of muscle mass loss.

### Bioimpedance parameters and phase angle measurements

Phase angle measurements demonstrated significant predictive value for muscle mass changes. Mean phase angle decreased from 4.8° ± 0.9° at baseline to 4.2° ± 0.8° at 12 months (*P* < 0.001). A strong correlation was observed between baseline phase angle and subsequent muscle mass loss (r = − 0.68, *P* < 0.001). ROC analysis revealed that a phase angle decrease of ≥ 0.3 degrees over three months predicted accelerated muscle loss with 86% sensitivity and 82% specificity (AUC = 0.89, 95% CI 0.83–0.95).

Changes in bioimpedance parameters showed distinct patterns across different measurement frequencies. The impedance ratio (Z200kHz/Z5kHz) demonstrated a progressive increase over the study period (mean change 0.084 ± 0.023, *P* < 0.001), indicating alterations in tissue conductivity and fluid distribution. These changes appeared earlier than the subsequent measurable decline in muscle mass, suggesting that alterations in electrical properties and fluid distribution precede detectable reductions in muscle quantity.

### Extracellular water distribution patterns

**Fig. 3:**
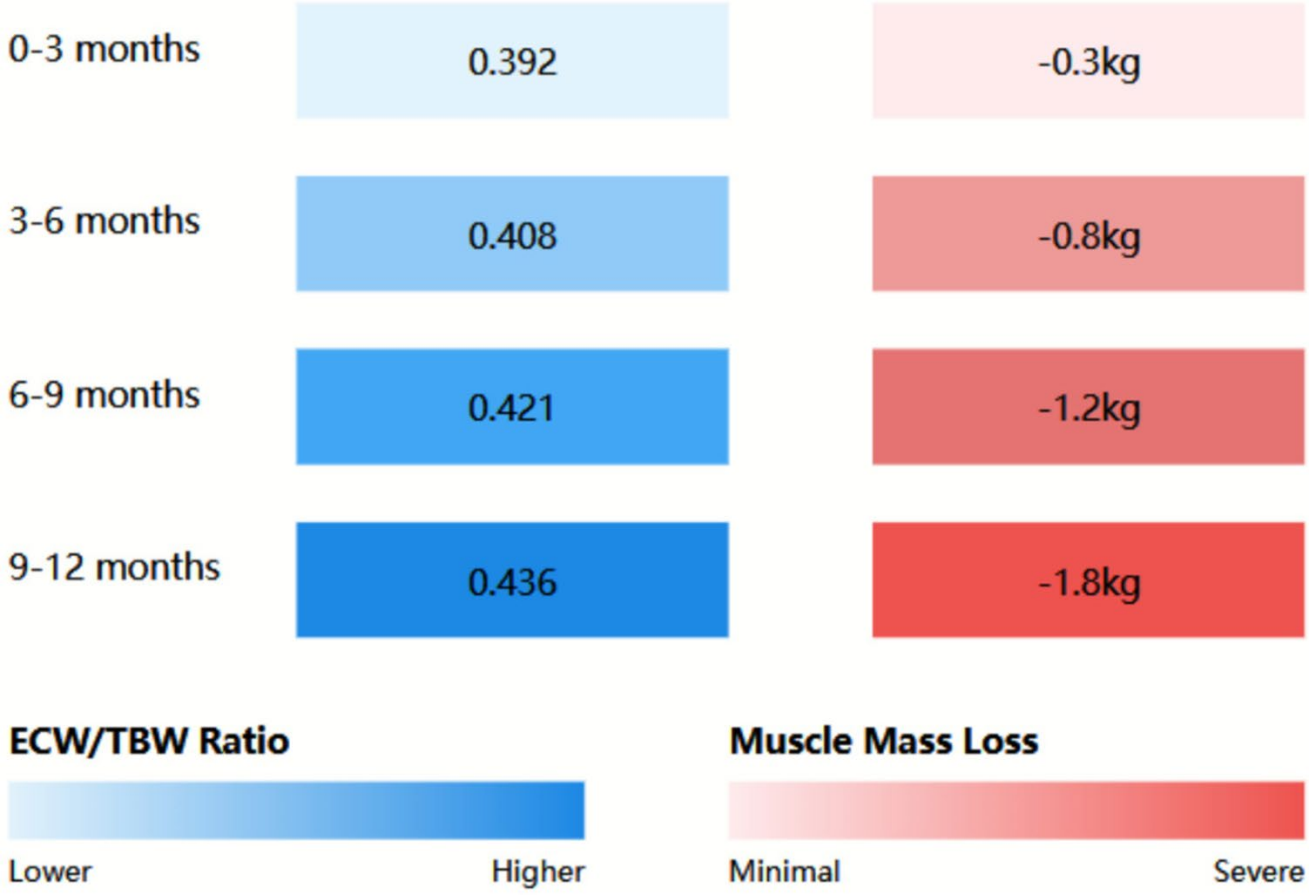
Relationship between ECW/TBW ratio and muscle mass loss heat map showing the relationship between extracellular water/total body water (ECW/TBW) ratio (blue gradient) and muscle mass loss (red gradient) over 12 months. Darker colors indicate higher values. Strong correlation was observed between ECW/TBW ratio and muscle mass loss (r = 0.72, *P* < 0.001)

Analysis of extracellular water (ECW) distribution revealed significant associations with muscle mass changes. The ECW/TBW ratio showed a progressive increase over the study period (mean change 0.024 ± 0.006, *P* < 0.001). This increase demonstrated a strong negative correlation with muscle mass (r = − 0.72, *P* < 0.001). Figure 3 illustrates the temporal relationship between ECW/TBW ratio changes and muscle mass loss.

Regional analysis of fluid distribution revealed heterogeneous patterns, with appendicular regions showing more pronounced changes compared to truncal areas. The mean difference in segmental ECW distribution between affected and unaffected muscle groups was 12.3 ± 3.4% (*P* < 0.001). These regional variations strongly correlated with localized muscle mass loss (r = 0.78, *P* < 0.001).

### Clinical correlations and functional outcomes

Functional parameters showed significant correlations with bioimpedance measurements. Handgrip strength demonstrated a positive correlation with phase angle (r = 0.65, *P* < 0.001) and an inverse correlation with ECW/TBW ratio (r = − 0.58, *P* < 0.001). Gait speed similarly correlated with phase angle measurements (r = 0.61, *P* < 0.001).

Inflammatory markers showed significant associations with both phase angle and ECW distribution. C-reactive protein levels inversely correlated with phase angle (r = − 0.54, *P* < 0.001) and positively correlated with ECW/TBW ratio (r = 0.49, *P* < 0.001). These relationships remained significant after adjusting for age, gender, and comorbidities.

### Subgroup analysis

Stratification by age revealed that participants over 75 years (n = 52) experienced more rapid muscle mass loss compared to younger participants (2.1 ± 0.7 kg vs. 1.6 ± 0.5 kg, *P* = 0.003). However, the predictive value of phase angle measurements remained consistent across age groups (interaction *P* = 0.418).

Gender-specific analysis showed similar patterns of association between bioimpedance parameters and muscle mass changes in both males and females, although absolute values differed significantly. The rate of phase angle decline was comparable between genders after adjusting for baseline values (*P* = 0.286).

Multiple regression analysis identified the following independent predictors of accelerated muscle mass loss: baseline phase angle (β = − 0.45, *P* < 0.001), ECW/TBW ratio changes (β = 0.38, *P* < 0.001), and inflammatory marker levels (β = 0.29, *P* = 0.002). These associations remained significant after adjusting for potential confounders including age, gender, and comorbidity burden.

## Discussion

This longitudinal study provides compelling evidence for the utility of combined multi-frequency bioelectrical impedance analysis and phase angle measurements in monitoring severe sarcopenia progression. Our findings demonstrate that changes in extracellular water distribution precede detectable muscle mass loss, offering potential early markers for disease progression and therapeutic intervention.

The observed temporal relationship between phase angle changes and muscle mass loss represents a significant advancement in understanding sarcopenia progression. Previous cross-sectional studies have suggested associations between phase angle and muscle function [23], but our longitudinal data demonstrate that phase angle changes precede clinically detectable muscle loss by approximately three weeks. This finding aligns with recent research indicating that cellular membrane integrity changes, reflected by phase angle measurements, may serve as early indicators of muscle quality deterioration [24, 25].

The strong correlation between extracellular water distribution patterns and subsequent muscle mass loss observed in our study provides new insights into the pathophysiological mechanisms of severe sarcopenia. Recent research has suggested that altered fluid distribution may contribute to muscle dysfunction through multiple pathways, including modified protein synthesis and degradation rates [26]. Our findings of regional variations in fluid distribution patterns, particularly in appendicular areas, support emerging evidence that fluid shifts may be both a marker and contributor to localized muscle deterioration [27, 28].

The predictive value of phase angle measurements demonstrated in our study (sensitivity 86%, specificity 82%) exceeds previously reported metrics for traditional sarcopenia monitoring methods. Recent meta-analyses of conventional techniques have reported sensitivities ranging from 67 to 78% for early detection of muscle mass changes [29]. The superior predictive capacity of phase angle measurements may be attributed to their ability to detect cellular-level changes before manifest tissue deterioration [30].

Our findings regarding the relationship between inflammatory markers and bioimpedance parameters provide important insights into the inflammatory component of sarcopenia progression. The observed correlation between C-reactive protein levels and phase angle measurements aligns with recent research suggesting that chronic inflammation may mediate changes in cellular membrane properties and fluid distribution [31]. These findings support the emerging concept of inflammation-mediated sarcopenia and suggest potential therapeutic targets [32].

The gender-specific differences observed in our study, particularly in the rate and pattern of muscle mass loss, contribute to the growing body of evidence suggesting sex-specific pathophysiological mechanisms in sarcopenia [33, 34]. Recent research has highlighted the role of hormonal factors and body composition differences in gender-specific sarcopenia progression [35, 36]. Our findings of comparable phase angle decline patterns between genders, despite different absolute values, suggest that the underlying cellular mechanisms may be similar despite phenotypic variations [37, 38].

The observed acceleration in muscle mass loss between months 3 and 6 of our study period provides important insights into the natural history of severe sarcopenia. This pattern aligns with recent longitudinal studies suggesting that sarcopenia progression may not be linear but rather characterized by periods of accelerated deterioration [39]. The ability to identify these high-risk periods through bioimpedance monitoring could enable more targeted therapeutic interventions [40].

The strong correlation between phase angle measurements and functional outcomes, including handgrip strength and gait speed, supports the clinical relevance of our findings [41]. Recent research has emphasized the importance of maintaining functional capacity in sarcopenia patients [42], and our results suggest that phase angle monitoring could provide early indicators of functional decline [43]. This association may be particularly valuable in clinical settings where regular functional testing is challenging or impractical [44, 45].

The age-stratified analysis in our study revealed important considerations for older persons populations. The more rapid muscle mass loss observed in participants over 75 years aligns with recent research suggesting accelerated sarcopenia progression in advanced age [46]. However, the consistent predictive value of phase angle measurements across age groups suggests that this monitoring approach remains reliable regardless of age [47].

Our findings regarding the ECW/TBW ratio changes provide new perspectives on fluid homeostasis in sarcopenia. Recent research has suggested that altered fluid distribution may reflect both systemic and localized tissue changes [48]. The strong correlation between ECW/TBW ratio changes and muscle mass loss observed in our study supports the use of this parameter as a monitoring tool and suggests potential therapeutic implications for fluid management in sarcopenia patients.

The limitations of our study warrant consideration. While our sample size was adequate for primary outcome analysis, larger studies may be needed to validate subgroup findings. Another potential limitation relates to the influence of hydration status on bioimpedance-derived parameters, particularly extracellular water distribution. Although all measurements were performed under standardized conditions (morning assessments after overnight fasting, post-voiding, and avoidance of caffeine, alcohol, and exercise), subtle intra-individual fluctuations in hydration cannot be entirely excluded. Such variations may contribute to minor measurement variability, especially in ECW/TBW ratios. Nevertheless, the consistent timing and environmental control applied across all visits are expected to minimize systematic bias, supporting the robustness of our longitudinal findings. Additionally, the 12-month follow-up period, while longer than many previous studies, may not capture the full spectrum of disease progression. While our multi-center design enhances generalizability, center-specific variations in care patterns may have influenced outcomes.

The clinical implications of our findings are substantial. The ability to detect impending muscle mass loss through bioimpedance monitoring could enable earlier intervention and potentially modify disease trajectory. Recent intervention studies have demonstrated that early therapeutic measures, including targeted exercise programs and nutritional supplementation, may be more effective when initiated before significant muscle mass loss. Our findings suggest that phase angle monitoring could help identify the optimal timing for such interventions.

Future research directions should include investigation of intervention strategies based on bioimpedance monitoring thresholds, development of predictive models incorporating multiple parameters, and exploration of the cellular mechanisms underlying the observed relationships. Additionally, studies examining the impact of various therapeutic interventions on bioimpedance parameters could provide valuable insights into treatment effectiveness.

## Conclusion

This longitudinal study demonstrates that multi-frequency bioelectrical impedance analysis combined with phase angle measurements provides a reliable method for monitoring severe sarcopenia progression. The strong correlation between extracellular water distribution changes and subsequent muscle mass loss, coupled with the high predictive value of phase angle measurements, offers a promising approach for early detection of disease progression. Changes in bioimpedance parameters preceded detectable muscle mass loss by approximately three weeks, potentially providing a critical window for therapeutic intervention. The observed associations between phase angle measurements, functional outcomes, and inflammatory markers suggest that this monitoring approach could have significant clinical utility.

## Data Availability

All data relevant to the study are included in the article or uploaded as online supplemental information. The datasets used and analysed during the current study are available from the corresponding author on reasonable request.

## Declarations

### Provenance and peer review

Not commissioned; externally peer reviewed.
